# A LATENT GROWTH CURVE MODEL OF RESOURCEFULNESS IN GRANDMOTHERS RAISING GRANDCHILDREN

**DOI:** 10.1093/geroni/igae098.2722

**Published:** 2024-12-31

**Authors:** Christopher Burant, Carol Musil, Jaclene Zauszniewski, Alexandra Jeanblanc

**Affiliations:** Case Western Reserve University, Cleveland, Ohio, United States; Case Western Reserve University, Cleveland, Ohio, United States; Case Western Reserve University, Cleveland, Ohio, United States; Case Western Reserve University, Cleveland, Ohio, United States

## Abstract

The role of grandmothers raising grandchildren has increased over the past three decades. Many of these grandmothers are living with their grandchildren and often these households do not include the parent of the grandchildren. Grandmothers raising grandchildren may deal with high levels of stress and strain that can contribute to high levels of depressive symptoms and potential mental health problems. Previous research has shown that resourcefulness can play a mediating role by reducing the impact of stress on mental health in caregiving situations. Resourcefulness comprises a cognitive behavioral skillset used for coping that includes cognitive reframing, problem-solving, and positive self-talk. Data were collected on 343 participants in a longitudinal nationwide online research study of caregiving grandmothers. In the current study, resourcefulness was assessed after grandmothers had received an intervention involving Resourcefulness Training© (RT) or journaling. A latent growth curve model was used to track the trajectory of resourcefulness at four time points (baseline, 2 weeks, 12 weeks, and 24 weeks) to identify whether resourcefulness improved over time. The model fit the data well (Chi Square=15.881; df=6; p=.014; TLI=.982; CFI=.989; RMSEA=.069). An additional multiple group nested model latent growth curve model found no differences between the 2 interventions. However, individuals receiving RT consistently had higher levels of resourcefulness and reached the maximum score at 3 months as compared to 6 months for those who journaled. Both interventions improved resourcefulness, indicating that interventions focusing on improving awareness of resourceful skills (e.g. problem-solving) may help grandmothers raising grandchildren with handling stress.

